# A graph-based genetic algorithm and generative model/Monte Carlo tree search for the exploration of chemical space†
†Electronic supplementary information (ESI) available: The codes used in this study can be found on GitHub: github.com/jensengroup/GB-GA/tree/v0.0 and github.com/jensengroup/GB-GM/tree/v0.0. See DOI: 10.1039/c8sc05372c


**DOI:** 10.1039/c8sc05372c

**Published:** 2019-02-11

**Authors:** Jan H. Jensen

**Affiliations:** a Department of Chemistry , University of Copenhagen , Copenhagen , Denmark . Email: jhjensen@chem.ku.dk ; http://www.twitter.com/janhjensen

## Abstract

This paper presents a comparison of a graph-based genetic algorithm (GB-GA) and machine learning (ML) results for the optimization of log *P* values with a constraint for synthetic accessibility and shows that the GA is as good as or better than the ML approaches for this particular property. The molecules found by the GB-GA bear little resemblance to the molecules used to construct the initial mating pool, indicating that the GB-GA approach can traverse a relatively large distance in chemical space using relatively few (50) generations. The paper also introduces a new non-ML graph-based generative model (GB-GM) that can be parameterized using very small data sets and combined with a Monte Carlo tree search (MCTS) algorithm. The results are comparable to previously published results (*Sci. Technol. Adv. Mater.*, 2017, **18**, 972–976) using a recurrent neural network (RNN) generative model, and the GB-GM-based method is several orders of magnitude faster. The MCTS results seem more dependent on the composition of the training set than the GA approach for this particular property. Our results suggest that the performance of new ML-based generative models should be compared to that of more traditional, and often simpler, approaches such a GA.

## Abstract 2

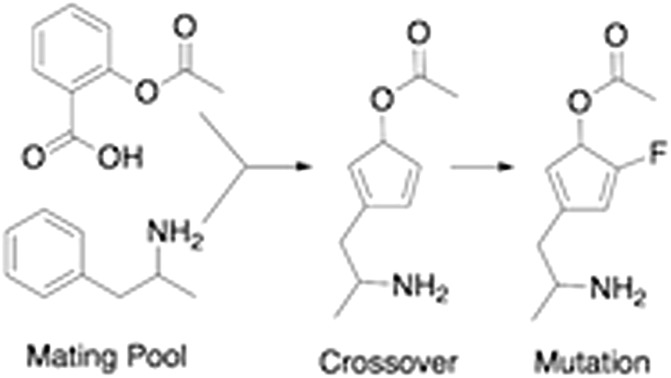
This paper presents a comparison of a graph-based genetic algorithm (GB-GA) and machine learning (ML) results for the optimization of log *P* values with a constraint for synthetic accessibility and shows that the GA is as good as or better than the ML approaches for this particular property.

## Introduction

Within the past few years several papers have been published on using machine learning (ML) to generate molecules with the aim of optimizing their properties.^1^–^8^ While the performances of these generative models have been impressive, they are usually not compared to those of more traditional approaches such as genetic algorithms (GAs),^9^–^12^ and recent work by Tsuda and co-workers^13^ suggests that non-ML approaches may be competitive. The lack of comparison is perhaps in part due to the fact that there are no free or open source versions of implementation of these GAs available (ACSESS^11^ uses the proprietary OpenEye cheminformatics toolkits).

In this paper I present a comparison of graph-based GA and ML results for the optimization of log *P* values with a constraint for synthetic accessibility and show that the GA is as good as or better than the ML approaches for this particular property. I also introduce a new non-ML graph-based generative model that can be parameterized using very small data sets and combined with a Monte Carlo tree search algorithm. The implementation of both methods relies on the open source RDKit cheminformatics package and the codes are made freely available.

## Computational methodology

### Graph-based genetic algorithm (GB-GA)

**Fig. 1:**
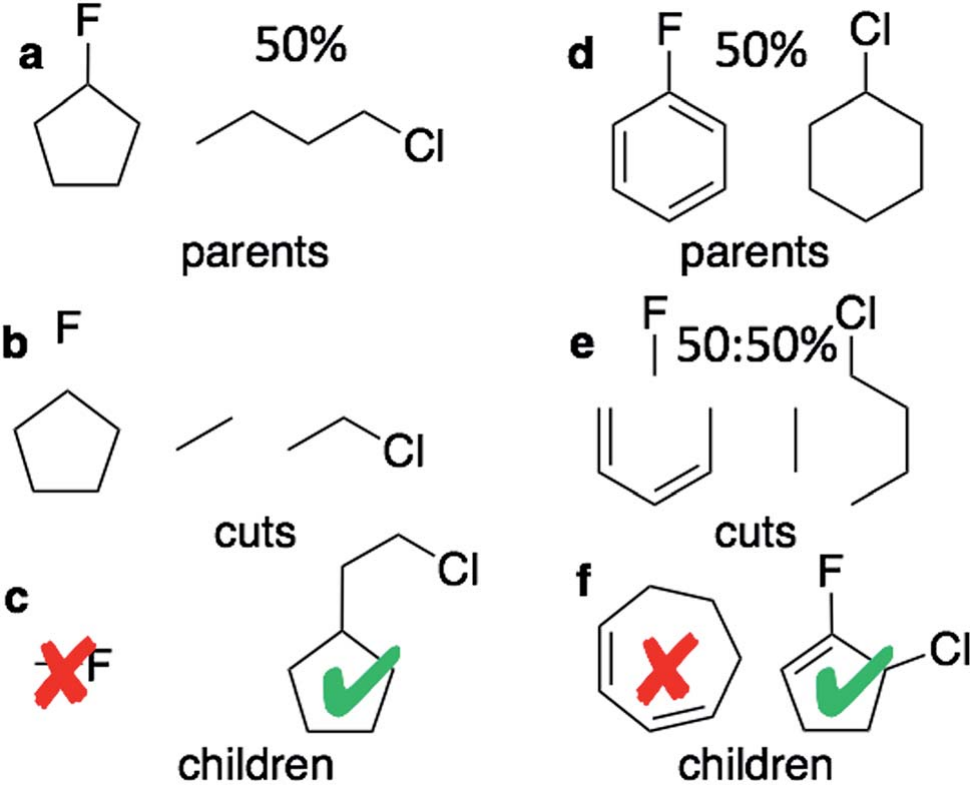
Two equally likely kinds of crossovers are considered: at non-ring (a–c) and at ring positions (d–f). Two equally likely kinds of ring cuts are considered: adjacent bonds and bonds separated by one bond. For ring crossovers fragments can be mated using both single and double bonds. (c) and (e) each shows two examples of children made by the mating process. Methylflouride is discarded because it is too small and the cycloheptene ring is discarded because the ring is too large.

**Fig. 2:**
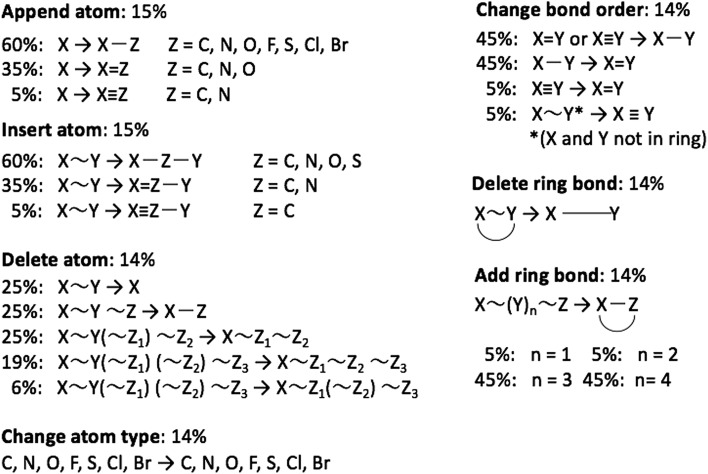
Overview of mutation operations and their associated probabilities, *e.g.* if an “append atom’’ mutation is chosen then a single bond is added 60% of the time.“∼” indicates an arbitrary bond order.

The graph-based genetic algorithm (GB-GA) combines the ideas from the algorithm developed by Brown *et al.*^9^ and the ACSESS algorithm developed by Virshup *et al.*^11^ and is implemented using the open source RDKit package. In this context “graph-based” means that crossovers and mutations are performed by altering a graph representation of the molecules as opposed to, say, a string based representation such as SMILES. The crossovers and mutations are defined using RDKit's reaction SMILES. Following Brown *et al.*^9^ a crossover can occur both at non-ring (Fig. 1a–c) and ring bonds (Fig. 1d–f), with equal probability and the positions of the cuts are chosen randomly. Molecules with macrocycles (seven atoms or more – Fig. 1f), allene centers in rings, fewer than five heavy atoms (Fig. 1c), incorrect valences as determined by RDKit, and more non-H atoms than the target size are discarded. The target size is a random number sampled from a distribution with a mean of 39.15 non-H atoms and a standard deviation of 3.50 non-H atoms as discussed later in the paper. The mutation operations are similar to those used by Virshup *et al.*^11^ (Fig. 2). The initial mating pool is drawn from a subset of the ZINC dataset used in previous studies,^2^,^4^,^6^ as described below, and molecules are sampled from the mating pool based on their normalized log *P* scores.

### A graph-based generative model with Monte Carlo tree search (GB-GM-MCTS)

Tsuda and coworkers^2^,^5^ have combined the text-based generative model developed by Segler *et al.*^1^ with a Monte Carlo tree search (MCTS) algorithm to optimize molecular properties. In this approach each character in a SMILES string corresponds to a node in a tree network and each node is selected sequentially using the MCTS approach. Inspired by Virshup *et al.*^11^ we developed a graph-based generative model (GB-GM) that grows the molecule one atom at a time and that can be combined with a MCTS. The GB-GM uses the “append atom” and “insert atom” mutations (Fig. 2) for atom addition to non-ring and ring atoms, respectively, but with different probabilities as described below. In addition to these two mutation-types, a new tricyclic ring-creation mutation is used: *X* ∼ *Y* → *X*1 ∼ *Z* ∼ *Y*1 where “1” indicates that *X* and *Y* are bonded and “∼” indicates an arbitrary bond order.

In order to create realistic looking molecules, such as those in the ZINC data set, the mutations and choice of element are chosen with probabilities obtained by an analysis of a subset of the molecules in the ZINC dataset. An analysis of first 1000 molecules in the ZINC dataset shows that 63% of the atoms are ring atoms, so the ring-creation or ring-insertion mutation is chosen 63% of the time. The most common 3-atom combination in rings is C=C–C, which accounts for 45% of all 3-atom combinations in rings (Table 1), so a C ∼ C → C=C–C mutation is chosen 45% of the time, and similarly for the 34 other *X* ∼ *Y* ∼ *Z* combinations found in the data set. The site of insertion/creation is chosen randomly and excludes aromatic and six-membered rings. Similarly, for addition the most common bond involving at least one non-ring atom is C–C, so a C → C–C mutation is chosen more often (see Table S1† for more details). A more specific bonding analysis, *e.g.* addition of C to C=C *vs.* C–C, was considered but then the most probable bonding patterns are often not found in the early stages of molecule growth and the growth process effectively stops.

**Table 1:** Probability of the 15 most common 3-atom combinations in rings in the first 1000 structures of the ZINC data set (“ZINC”), and in the 1000 structures generated by the GB-GM method using the ZINC probabilities (“GB-GM (62%)”) and a probability set where the probability of [*]=[*]–[*] type bonding is increased to 80% (“GB-GM (80%)”)

Bonding	ZINC	GB-GM (62%)	GB-GM (80%)
C=C–C	45%	41%	53%
C–C–C	15%	23%	21%
C–C–N	9%	9%	6%
C–N–C	6%	7%	5%
C=C–N	4%	6%	4%
N=C–C	3%	2%	2%
C=N–C	2%	2%	1%
C–C–O	2%	2%	2%
N=C–N	2%	0%	0%
C=N–N	2%	0%	0%
C–O–C	1%	1%	1%
C–N–N	1%	1%	1%
C=C–S	1%	1%	0%
C–S–C	1%	1%	1%
C=C–O	1%	1%	1%

The GB-GM-MCTS code is a modified version of the mcts.py code^14^ modified for leaf parallelization with a maximum of 25 leaf nodes. The exploration factor is 
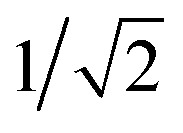
 and rollout is terminated if the molecule exceeds the target size as described for the GB-GA. Any three- or four-membered alkene rings are subsequently expanded to five-membered rings by inserting C atoms. The reward function is 1 if the predicted *J*(m) value (see below) is larger than the largest value found so far and 0 otherwise. This reward function was found to work slightly better than the one used by Yang *et al.*^2^

### The penalized log *P* score

Following Gómez-Bombarelli *et al.*^4^ and Yang *et al.*^2^ the goal is to maximize *J*(m):

1 *J*(m) = log *P*(m) – SA(m) – RingPenalty(m)

where log *P*(m) is the octanol–water partition coefficient for a molecule (m) as predicted by RDKit, SA(m) is a synthetic accessibility score,^15^ and RingPenalty(m) penalizes unrealistically large rings by reducing the score by RingSize – 6 where RingSize is the number of atoms in a ring. Following previous studies, each property is normalized to have zero mean and unit standard deviation across the ZINC dataset.

*J*(m) depends both on the number and types of atoms and can be made arbitrarily large by increasing the molecular size. Therefore it is important to limit the molecular size in order to make a fair comparison to previous studies. Yang *et al.*^2^ predict SMILES strings with a maximum length of 81 characters, but it is difficult to translate that restriction directly to molecular size since many of the characters do not refer to atoms. Instead, the 20 molecules found by Yang *et al.*^2^ are used to determine an average target size of 39.15 ± 3.50 non-H atoms.

## Results and discussion

### GB-GA

Ten GA simulations are performed and the maximum *J*(m) scores for each simulation are averaged. The population size is 20 and 50 generations are used (*i.e.* 1000 *J*(m) evaluations per run). The initial mating pool is 20 random molecules sampled from the first 1000 molecules in the ZINC data set. The mean *J*(m)-score for this set is 0.2 and the maximum value is 3.6.

The average maximum *J*(m)-score for the GA is 6.8 ± 0.7 and 7.4 ± 0.9 using a 50% and 1% mutation rate, respectively (Table 2). For comparison the best average maximum value found by Yang *et al.*^2^ is 5.6 ± 0.5, which required close to 20 000 *J*(m)-score evaluations. The latter took 8 hours each, while the GB-GA calculations took 30 seconds each on a laptop. These *J*(m)-scores are also significantly larger than those of the other ML-based methods tried by Yang *et al.*^2^ (Table 2): a recurrent neutral network (RNN) with and without Bayesian optimization (BO), the continuous variational autoencoder^4^ (CVAE), and the grammar variational autoencoder^3^ (GVAE).

**Table 2:** Maximum *J*(m) scores averaged over 10 runs, the number of molecules evaluated per run, and the required CPU time per run. See the text for an explanation of the methods. Results for the non-GB methods are taken from the study of Yang *et al.*^2^ where the number of molecules evaluated per run is estimated based on the average number of molecules generated per minute and the CPU time

Method	Average *J*(m)	No. molecules	CPU time
GB-GA (50%)	6.8 ± 0.7	1000	30 seconds
GB-GA (1%)	7.4 ± 0.9	1000	30 seconds
GB-GM-MCTS (62%)	2.6 ± 0.6	1000	90 seconds
GB-GM-MCTS (80%)	3.4 ± 0.6	1000	90 seconds
GB-GM-MCTS (80%)	4.3 ± 0.6	5000	9 minutes
ChemTS	4.9 ± 0.5	∼5000	2 hours
ChemTS	5.6 ± 0.5	∼20 000	8 hours
RNN + BO	4.5 ± 0.2	∼4000	8 hours
Only RNN	4.8 ± 0.2	∼20 000	8 hours
CVAE + BO	0.0 ± 0.9	∼100	8 hours
GVAE + BO	0.2 ± 1.3	∼1000	8 hours

**Fig. 3:**
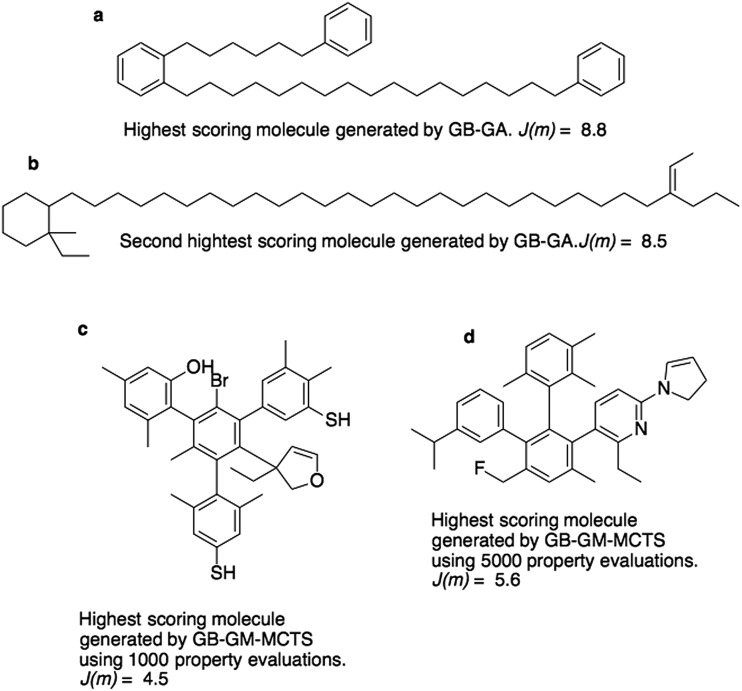
Highest scoring molecules from the GB-GA (a and b) and GB-GM-MCTS (c and d) searches.

Fig. 3a and b show the molecules with the two highest *J*(m)-scores found by the GB-GA. These scores, 8.8 and 8.5, are slightly larger than the three largest values (7.8–8.0) obtained by You *et al.*^6^ using a graph convolutional policy network approach. The two molecules bear little resemblance to the molecules used to construct the initial mating pool. The molecules in the ZINC data set that are most similar to these two molecules have respective Tanimoto similarity scores of just 0.27 and 0.12 and corresponding *J*(m) values of 0.9 and –2.4 (Fig. S1†). This indicates that the GB-GA approach can traverse a relatively large distance in chemical space using relatively few (50) generations.

**Fig. 4:**
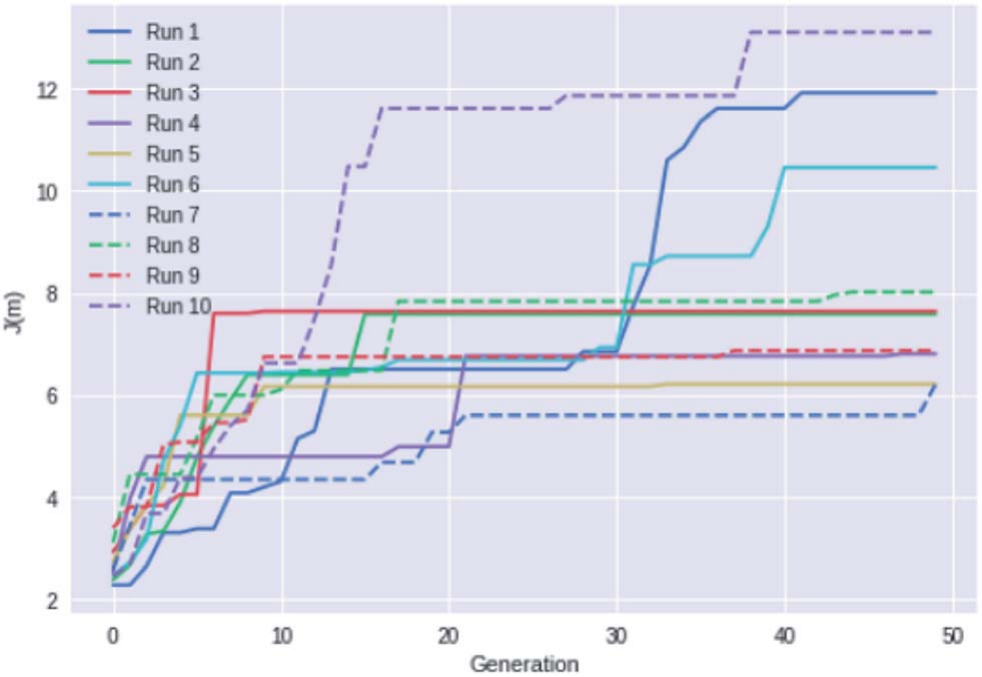
Plot of the highest *J*(m) value found as a function of generations for 10 different GB-GA runs with a mutation rate of 1%.

Fig. 4 shows an example of how the maximum *J*(m) value evolves with each generation for 10 different GB-GA runs. While most of the runs have mostly peaked after about 20 generations the three best performing runs (run 1, 6, and 10) show significant improvements between 30 and 40 generations, so running fewer than 50 generations cannot be recommended for *J*(m) maximisation. None of the runs increased *J*(m) significantly after periods of 20 generations with no or little change in *J*(m) (with the possible exception of run 7). So a good strategy may be to terminate the GB-GA run if the *J*(m) value has not changed for more than 20 generations (at least for *J*(m) maximisation).

### GB-GM

As described in the Computational methodology section, the GB-GM method grows a molecule one atom at a time where the choice of bond order and atom type is chosen probabilistically based on a bonding analysis of the first 1000 molecules in the ZINC dataset (referred to hereafter as the reference set). The GB-GM model is tested by generating 1000 molecules using ethane as the “seed” molecule (which takes about 30 seconds on a laptop) and repeating the statistical bonding analysis. The average molecular size in the new data set is 23.2 ± 4.1 atoms, which is nearly identical to that of the training set: 23.2 ± 4.4 atoms. There are 2498 rings compared to 2768 in the reference set and 59% of the atoms are in rings, which also is close to the 63% in the reference set. 41% of the 3-atom combinations in rings is C=C–C, which is reasonably close to the 45% in the reference set (Table 1). This difference is probably due to the fact that at least one of the two carbons must be secondary to “accept” the double bond, and if such a bonding pattern is not present then no “=C–” atom will be added. It is thus a little bit more likely that a “–C–” atom will be “accepted”, compared to a “=C–” atom.

Not surprisingly, there are bigger differences for the larger scale features not specifically encoded in the rules such as the type of ring (Table 3). For example, there are many fewer benzene-type rings ([*]1=[*]–[*]=[*]–[*]=[*]1) as well as cyclopentane ([*]1–[*]–[*]–[*]–[*]1) and cyclohexane ([*]1–[*]–[*]–[*]–[*]–[*]1) type rings, while there are more of most of the other types compared to the reference set. The reason is that the molecules in the ZINC data set are not made by randomly adding atoms, but by assembling larger functional groups such as aliphatic and aromatic rings. As a result the average ring composition does not reflect the most likely ring compositions. It is possible to scale the probabilities to skew the results towards one or the other ring-type. For example in the last column the probabilities are scaled such that the probability of *X* = *Z*–*Y* is 80% rather than the 62% in the reference set, which increases the number of benzene-like rings from 479 to 850 at the expense of the aliphatic rings.

**Table 3:** Number of occurrences of different ring-types in the first 1000 structures of the ZINC data set (“ZINC”), and in the 1000 structures generated by the GB-GM method using the ZINC probabilities (“GB-GM (62%)”) and a probability set where the probability of [*]=[*]–[*] type bonding is increased to 80% (“GB-GM (80%)”)

Ring-type	ZINC	GM (62%)	GM (80%)
[*]1–[*]–[*]1	57	104	57
[*]1–[*]–[*]–[*]1	17	33	10
[*]1–[*]–[*]–[*]–[*]1	280	15	4
[*]1=[*]–[*]–[*]–[*]1	120	396	408
[*]1=[*]–[*]=[*]–[*]1	470	132	221
[*]1–[*]–[*]–[*]–[*]–[*]1	409	64	2
[*]1=[*]–[*]–[*]–[*]–[*]1	77	363	104
[*]1=[*]–[*]=[*]–[*]–[*]1	100	591	405
[*]1=[*]–[*]–[*]=[*]–[*]1	7	321	202
[*]1=[*]–[*]=[*]–[*]=[*]1	1206	479	850
7-Membered ring	24	0	0
8-Membered ring	1	0	0
Total	2768	2498	2263

### GB-GM-MCTS

Ten GB-GM-MCTS simulations are performed using ethane as a seed molecule and the maximum *J*(m)-scores for each simulation are averaged. The tree is traversed 1000 times, *i.e.* there are 1000 *J*(m) evaluations per run. For GB-GM-MCTS the average maximum *J*(m)-score value is 2.6 ± 0.6, which is significantly lower than the lowest value in the study of Yang *et al.*^2^, 4.9 ± 0.4 (Table 2). The most likely explanation is that the GB-GM makes relatively few benzene rings (as discussed above), which, together with Cl atoms, is the defining structural feature of the high scoring molecules found by Yang *et al.*^2^ Indeed, if the probability of generating C=C–C containing rings is increased from 62% to 80% then the average maximum *J*(m)-score increases to 3.4 ± 0.6. Increasing the number of tree traversals to 5000 increases the value to 4.3 ± 0.6, which is similar to the 4.9 ± 0.4 obtained by Yang *et al.*^2^ using as similar number of tree traversals. The latter run takes about 9 minutes, compared to 2 h in the study of Yang *et al.*^2^.

Fig. 3c and d show the highest scoring molecule found using 1000 and 5000 tree traversals. The molecules are similar to those found by Yang *et al.*^2^ in that they consist mostly of benzene rings, and the benzene ring is the most common hydrophobic structural motif in the ZINC data set. The GB-GA results show that higher *J*(m)-scores can be obtained using long aliphatic chains, but this structural motif is relatively rare in the ZINC data set and therefore rarely suggested by the generative models.

## Conclusion and outlook

This paper presents a comparison of a graph-based genetic algorithm (GB-GA) and machine learning (ML) results, compiled by Yang *et al.*,^2^ for the optimization of log *P* values with a constraint for synthetic accessibility (*J*(m)) and shows that the GA is as good as or better than the ML-based approaches for this particular property. The GB-GA predicts maximum *J*(m)-values that, on average, are 1.3–1.8 units higher than the best ML-based results reported by Yang *et al.*,^2^ while also being several orders of magnitude computationally more efficient. Similarly, the GB-GA method finds molecules with maximum *J*(m)-scores that, depending on the method, often are several units larger than those found with ML-based approaches. These molecules bear little resemblance to the molecules used to construct the initial mating pool, indicating that the GB-GA approach can traverse a relatively large distance in chemical space using relatively few (50) generations.

The paper also introduces a new non-ML graph-based generative model (GB-GM) that can be parameterized using very small data sets and combined with a Monte Carlo tree search (MCTS) algorithm such as the one used by Yang *et al.*^2^ The results are comparable to the results obtained by Yang *et al.*^2^ using a recurrent neural network (RNN) generative model, with maximum *J*(m)-values of 4.3 ± 0.6 compared to 4.9 ± 0.6 found using 5000 property evaluations. While the results are slightly worse than the RNN results, the GB-GM-based method is several orders of magnitude faster. In both cases the MCTS approach essentially extracts the main hydrophobic structural motif (a benzene ring) found in the training set. The MCTS results thus seem more dependent on the composition of the training set than the GA approach for this particular property.

While the results are quite encouraging, it is important to perform similar comparisons for other properties before drawing general conclusions. In a very recent study Brown *et al.*^16^ have compared GB-GA and GB-GM-MCTS methods to SMILES-based ML and GA methods on 20 different optimization problems and conclude that “In terms of optimization performance, the best model is a genetic algorithm based on a graph representation of molecules”. Our and their results strongly suggest that the performance of new ML-based generative models should be compared to that of more traditional, and often simpler, approaches such as GAs. Both the GB-GA and GB-GM-MCTS codes used in this study are freely available with the open source RDKit toolkit as the only dependence.

## Conflicts of interest

There are no conflicts to declare.

## Supplementary Material

Supplementary information
