# Supplementary material for: A graph-based genetic algorithm and generative model/Monte Carlo tree search for the exploration of chemical space
Source: Chem Sci. 2019 Feb 11;10(12):3567–72. doi: 10.1039/c8sc05372c (PMC6438151; doi:10.1039/c8sc05372c)
Supplement: Supplementary file 1 [file SC-010-C8SC05372C-s001.pdf]

This journal is © The Royal Society of Chemistry 2019

SUPPORTING INFORMATION

Table S1. Probability of a bonds, where the second atom is not in a ring, in the first 1000 molecules in the ZINC dataset.

bond	prob.	bond	prob.
C-C	37.87%	C≡N	0.41%
C-N	14.07%	N-N	0.33%
N-C	13.76%	N-O	0.31%
C=O	8.28%	S-N	0.30%
C-O	7.96%	O-N	0.29%
O-C	4.83%	N=O	0.25%
C-S	2.27%	C=N	0.23%
C-F	2.23%	C=S	0.18%
S=O	1.62%	N=C	0.15%
C-Cl	1.44%	C≡ C	0.12%
S-C	1.10%	S=C	0.06%
C=C	0.91%	O-S	0.02%
N-S	0.56%	S-O	0.02%
C-Br	0.41%	C-I	0.01%

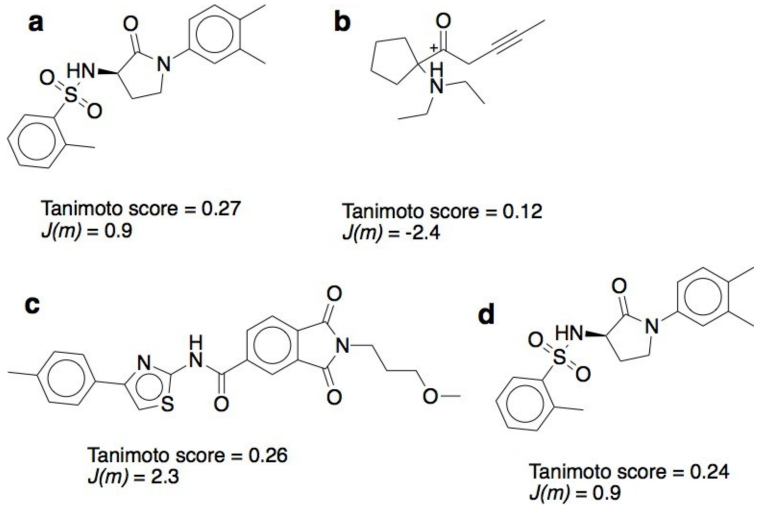

Figure S1. Molecules from the ZINC data set with the highest Tanimoto similarity to the molecules shown in Figure 3, together with their $J(m)$ values. The Tanimoto score is calculated using ECFP4 fingerprints.
